# A Legal Jurisprudential Deliberation on Lineage and Inheritance of the Pre-Implantation Embryo

**Published:** 2012-03-20

**Authors:** Mohammad Taqi Rafiei

**Affiliations:** Department of Private Law, Faculty of Law, University of Tehran , Tehran, Iran

**Keywords:** Artificial Insemination, Pre-implantation Embryo, Lineage, Inheritance

## Abstract

Today, *in vitro* fertilization (IVF) is not just a medical issue, but certainly also a complex legal issue, in which lawyers can play an important role by establishing the suitable legal conditions to regulate legal relations by introducing necessary theories. One of the important and controversial issues, which can be approached legally, is the study of the pre-implantation embryo with regard to property law, especially the inheritance of the pre-implantation embryo. Article 3 of the Conditions of Embryo Donation to Infertile Couples Act of the year 2003 only stipulates the responsibilities of the intended couple and born child in terms of support, upbringing, maintenance and respect; it does not specify any law regarding the other financial outcomes of lineage like "inheritance", which makes this law imperfect. Also, in view of the fact that lineage is one of the causes of inheritance; studying inheritance without analyzing lineage in the pre-implantation embryo is not possible. Therefore, it is recommended to study lineage and inheritance simultaneously. Some questions arise in this regard, including whether it is possible to prove lineage between the genetic father and mother with a laboratory child, and between the owner of the womb (that is intended wife) and the child. Supposing the lineage is proved, what is the state of inheritance between them? Lawyers and Islamic jurists have different opinions regarding the lineage of the pre-implantation embryo and inheritance. The author believes that the owner of the sperm is regarded as the genetic father of the child and in terms of lineage between laboratory child and mother two genetic and carrying factors must be considered. Thereby, considering inheritance between the genetic father and the child is possible according to inheritance law. Regarding the inheritance state of a laboratory child from two mothers the problem can be solved by using the equality rule within the framework of inheritance law.

## Introduction

The pre-implantation embryo, as the product of human reproduction process through unconventional methods and as the result of advancement in human knowledge, raises legal issues as well as carrying medical aspects and social consequences. The inevitability of this technology has led jurists to provide appropriate solutions to regulate the legal aspects of this complex phenomenon and also to prove their modern ideas with regard to the old legal structure and codify new laws according to the requirements of time and place.

Among various legal issues in this regard, studying the pre-implantation embryo in the field of property law, especially the pre-implantation embryo’s inheritance, is considered an important issue. In spite of the innovations and legal solutions provided, the Conditions of Embryo Donation to Infertile Couples Act, year 2003 in Iran (1), has not stipulated any rule regarding lineage itself as well as the financial outcomes of lineage, like inheritance. Article 3 of this Act, which only stipulates the responsibilities of intended couples and born child, has added some ambiguities in terms of inheritance. This article will attempt to compensate for such a lack of regulations by outlining related issues and providing some suggestions.

According to Iranian law, lineage is one of the important causes of basis for inheritance (2). The lineage resulting from artificial insemination is certainly a complex legal issue and in this regard there are various opinions. This research investigates the related issues of lineage and inheritance our society is faced with. I discuss, at first, the lineage of the pre-implantation embryo and then the related issues of inheritance. It should be noted that infertility is sometimes the result of weakness or lack of the husband's sperm, which is cured through sperm donation, or the result of weakness or lack of the wife's ovule, which is cured through ovum donation, and sometimes related to the couple and is cured through embryo donation. This article studies lineage and inheritance in the area of embryo donation. I also remind that the artificial insemination is carried out through different methods; this article only emphasizes the embryo transfer resulting from in-vitro fertilization or in other words, the pre-implantation embryo, since the above mentioned Act only stipulates this method of embryo transfer.

### Part 1: The lineage of pre-implantation embryo

As the lineage is one of the inheritance causes according to Iranian law, the inheritance of the pre-implantation embryo is undoubtedly dependent on proof and disproof of the lineage. However the legal status of a laboratory child is an important legal issue, because the lineage and its proof are matters of utmost importance. The Holy Qur'an, in Surat Al-Furqan, verse 54, hints at the importance of human lineage (kindred of blood): *"And it is He who created of water a mortal, and made him kindred of blood and marriage …"* (3). In this regard, issues like the followings arise: Is the born child legitimate or illegitimate? In case of legitimacy, to whom is its lineage attributed? Since the legitimacy of such fertilization and the laboratory child on the Islamic jurisprudential bases and the legal principles, is not the subject of discussion here, we will focus on the issue of the lineage on which the inheritance is based. For this purpose, the concept of lineage, the conditions of achievement of legitimate lineage and finally the pre-implantation embryo's father and mother are studied.

### The concept of lineage

'Lineage', literally, means relationship. Other meanings of lineage are: origin, race and connection between two entities (4). It also means attributing a person to another person (5). In French, “*filiation*” means dynasty, generation, connection and pursuance of the things resulting from one another (6). Adapted to the common understanding, a child resulting from the sexual intercourse of a man and a woman forms a relationship among itself and its parents; this relationship is called 'lineage'. Some Islamic scholars have indicated: "lineage means the birth of a person resulting from another person, like the father and the son; or the birth of two people (like two brothers) resulting from a third party (mother or father)" (7).

With regard to the legal nature of lineage, it must be noted that although the first part of the eighth book of the Iranian Civil Code is dedicated to commandments related to lineage, there is no exact definition of its nature. Therefore, the definitions provided by lawyers and Islamic scholars are discussed.

Some lawyers have defined lineage in this way: "Lineage means relationship and is achieved through formation of zygote resulting from sexual intercourse of a man and a woman. This arises from the natural and blood relationship between the child and other two people who are his parents" (8). Some other lawyers indicate: "lineage is the bond between two people, which happens as the result of birth of one of them from the other or the birth of both of them from a third party" (9). Some criticize the definitions provided by lawyers and Islamic scholars on the basis that ambiguous words have been used, but the purpose of legislation by lineage is its common understanding. Finally, they indicate: "From both the common understanding and literal aspects, lineage is a relative term which is realized through creation of a human being from the gamete of other human being" (10).

Some lawyers believe that lineage in the specific meaning is a natural and blood relationship between two people one of whom is created directly from the other’s sperm or ovum (11). The basis of lineage realization and the main origin of connection of the child to the male and the female is that very formation of the zygote, and this, today, is adapted to the common understanding. This main origin has been ignored in the Islamic rules only in the case of fornication. Therefore, childbirthresulting from fornication hinders the realization of legitimate lineage. Otherwise, the natural lineage must be regarded as legitimate and valid, like lineage resulting from doubt. According to Articles 1156 and 1166 of the Iranian Civil Code, the main criterion of connecting lineage between a child and a person or persons is its creation from their gametes. But, the Iranian legislator regards fornication as the only hindrance of realization of legitimate lineage in Article 1167 of the Civil Code (12). Therefore, in cases not involving fornication, there is no hindrance to the connection of the child to the person whose gamete has brought it into existence (13).

Therefore, as an accepted definition of lineage in this article, we can say that lineage is a relative term which is realized through the creation of a human being from the gamete of another human being, and this concept of lineage is accepted by general custom.

### The conditions of achievement of legitimate lineage

In order to prove the connection of legitimate lineage of a child, the child's father and mother must be married to each other, thus the connection of a child to his father and mother is realized. If a zygote is formed as a result of sexual intercourse and a man and woman get married afterwards and childbirth happens when they are a couple, the child lineage is not legitimate. Therefore, according to Articles 1158 and 1167 of the Civil Code (12), it can be said that the requirement of realization of legitimate lineage is the formation of the zygote at a time when the man and woman are married and this condition is supported by the established and normative usage, which is based on Islamic jurisprudence.

Some lawyers have analyzed the conditions of the realization of legitimate lineage, so they consider it in two parts. Firstly, lineage realization through marriage relationship. That is, a child is legitimate if its parents have a legitimate marriage and the child is the woman’s legitimate child if it is her husband’s child too. And the legitimate lineage of the child to the man is realized if the child is considered as his wife’s child too. Secondly, lineage out of family bond, as a result of doubtful sexual intercourse. That is, the child may have a different father or mother. In the event that a married woman mistakenly has sexual intercourse with a stranger, the child resulting from this relationship is the woman's child, not her husband's, and vice versa.

Based on what is mentioned about the motherhood and fatherhood lineage relation, the common fundamentals of these two lineages within family are summarized as follows:

1. A man and a woman whose lineage to a child is being discussed must be husband and wife.
2. The childbirth must have been happened as a result of their sexual intercourse.
3. Sexual intercourse and the formation of a zygote must have been happened after their marriage (14).

In terms of lineage realization out of family, it must be indicated that based on Islamic jurisprudence and the laws originated from that, in some cases a child created out of family is connected to its parents too, and so such a lineage is regarded as legitimate, as follows:

1. Doubtful sexual intercourse, by those who as a result of ignorance of law or fact, were not aware of sexual intercourse of the nullity of marriage.
2. Sexual intercourse by those who have no discriminative faculty.
3. Sexual intercourse by those who have been forced under duress.
4. A child resulting from artificial insemination of a woman by the sperm of a stranger (15).

Thereby, we can conclude that according to the Islamic jurisprudential bases and legal principles, the pre-implantation embryo's lineage or laboratory child's lineage is one of the above mentioned cases considered out of family that can be connected to the persons who play a role in its creation.

### The pre-implantation embryo's father and mother

Different people play roles in the creation of a child resulting from artificial insemination but the most important question is who are the parents of a child resulting from embryo donation? It is eviLineagedent that as inheritance is based on this matter, first, the parents of a pre-implantation embryo must be identified. Thus, here the fatherhood and motherhood lineage of such a child are studied.

### The fatherhood lineage

Based on medical science, undoubtedly the origin of the embryo on the father's side is sperm. Moreover, it is commonly understood that the creation of one from the other is a criterion for lineage and relationship between two people and thereby, realizes 'lineage' to describe such a real and genetic event. The verses of the Holy Qur'an (3) and jurisprudential rules, which will be mentioned later, as well as the laws of most countries of the world, support this idea. With slight differences, the laws of most countries indicate this real and natural relationship as lineage, and therefore recognize various effects on this relationship and lineage.

As you might know, the proof of the fatherhood lineage has been mentioned in Articles 1158 to 1167 of the Iranian Civil Code. For instance, Article 1158 of the Civil Code states: "Any child born during marriage belongs to the husband provided that the interval between intercourse and the birth of the child is not less than 6 months and not more than 10 months". What is understood from this Article and its following Articles indicates that the criterion for determination of the fatherhood lineage is the time of zygote formation. The time of zygote formation is very important because normally the time of the meeting of man's sperm and woman's ovum is not clear even for the couple. Therefore, the Iranian legislators attempt to create a presumption called 'presumption of legitimacy', which partly in cases of doubt determines the real lineage.

Therefore, based on this rule and regarding the stipulated conditions in Article 1158 of the Iranian Civil Code, if childbirth happens during the marriage, the presumption is that the child has been created from the husband's sperm so is connected to him, unless it is proved that this child is created from another man's sperm. It is noted that according to some Islamic jurists as Ayatolla Khoei (16) and Ayatolla Sistani (17), considering the condition of 'legitimate intercourse,' the lineage of the created child resulting from fornication is not connected to the fornicator.

Consequently, according to the mentioned definition of legitimacy, in the view of some lawyers, "the criterion is the legitimate sexual intercourse between the couple, and this intercourse in its broad sense includes laboratory insemination too" (10). Sexual intercourse has been provided as a cause of zygote formation in Article 1158 of the Civil Code, and the sperm and ovum can also meet in the laboratory insemination procedure, creating a child. That is, if the husband's sperm and the wife's ovum meet outside of the wife's womb, the embryo formed is connected to the mentioned couple, though they had no sexual intercourse. Thereby, such insemination is included in Article 1158 of the Civil Code, and the presumption of legitimacy is applicable to that too.

Some other lawyers who support the above idea indicate: "marriage is not enough for establishment of blood relationship between child and his parents. The thing which naturally and by blood forms blood relationship as father, mother and child is the sexual intercourse between the father and the mother. Marriage is the condition for lineage legitimacy, and such condition is effective only when sexual intercourse, as the actual cause has happened. Here, the sexual intercourse does not have a conventional meaning but the point is that the embryo has been created through act of mixing man and woman's gametes. Though the conventional meaning of sexual intercourse is what happens mostly" (18). The author believes that this is also true about the state of the embryo resulting from artificial insemination. That is, what matters is that the child is created from a mixture of the legitimate husband and wife's gametes. The necessity of 'the condition of sexual intercourse' is that the gametes of both legitimate husband and wife are placed near each other, whether naturally or artificially. Thus, the sexual intercourse of the couple in a general sense (not specific to the conventional meaning) includes both the natural form and artificial insemination. And to achieve the condition for lineage legitimacy, the mixture of the couple's gametes shall be related to the marital relation.

Regarding the fatherhood lineage it is important to note that as artificial insemination is included in Article 1158 of the Civil Code, the pre-implantation embryo has been created from the husband's sperm so is connected to him. Also, a large number of scholars (19, 20) do not have any doubt in the connection of lineage of the pre-implantation embryo to the owner of the sperm. Of course, some Islamic scholars, like Ayatollah Hakim, believe that in the realization of the lineage two factors are effective: the first, legitimate sexual intercourse between the couple and second, sperm transfer through conventional methods. Therefore, if the sexual intercourse between a man and a woman is not legitimate, lineage between them and the child will not be established, even if the sperm transfer happens through conventional methods. Moreover, if the intercourse between a man and a woman is legitimate but the sperm transfer is performed through unconventional methods, lineage will not be established.

However, such theory is not supported by solid jurisprudential reasoning, because as mentioned above many Shiite scholars believe that the embryo belongs to the owner of the sperm, even if the sperm transfer is performed through forbidden deeds like lesbianism (21-23). The genuine Traditions (Hadith) also support this theory, as is shown by the Tradition attributed to Imam Hasan (the second Imam and religious leader of Shiites) about lesbianism of a virgin with a married woman (24). Thereby, according to what is mentioned, it is concluded that the sperm donor has been recognized as the father.

### The motherhood lineage

There are different opinions regarding the determination of motherhood lineage, which is discussed here.

### First opinion: the owner of womb is the real mother

Followers of this theory believe that at the time of the revelation of the Holy Qur'an and the expression of Traditions, people had no knowledge about new medical information that the child is as the result of fertilization of sperm and ovum. And they thought of the womb as a place for embryo growth, so they considered the owner of womb as the mother. This indicates that the basis of common judgment was childbirth. i.e., they considered the woman who gives birth to a child as its mother; thus the Islamic legislator supported this common judgment under a few conditions (25). Supporting this theory, some Islamic scholars such as Ayatolla Moosavi Bojnoordi and Ayatolla Tabrizi (26) and Ayatolla Khoei (16) believe that the legal mother of a child resulting from such process is the woman who has given birth to him/her after pregnancy termination.

This group relies on some verses of the Holy Qur'an like Surat Al-Ahqaf, verse 15 and Surat Al-Luqman, verse 14, to support their theories. The first and the most important verse is Surat Al-Mojadele, verse 2: *"those of you who say, regarding their wives, Be as my mother's back, they are not truly their mothers; their mothers are only those who bore them"* (3). That is, their mothers are only those mothers who give birth to the child. This verse is used on the basis that it considers a woman who gives birth to a child as mother, whether the ovum is hers or not,especially since it expresses exclusivity with words like *"their mothers are only"*. Though here, the contents of the verse is related to the 'injurious comparisons' case, i.e.; a kind of repudiation of the marital relationship among pre-Islamic Arabs which took place on a husband's saying to his wife "Be as my mother's back" (zahr, hence the derivative zihar) (27), and the verse rejects those who considered their wives as their mothers. But the followers of this view believe that it is proved in knowledge of the principles of jurisprudence, and in the eyes of the wise persons ostensible meaning of the verse is a criterion for validity. Thus, the group relying on the mentioned verse believes that only the woman who gives birth to a child is the mother.

However, some scholars have questioned relying on the above verse in this regard because *"who bore them"* in this verse does not mean giving birth to a child, as in some Traditions this phrase has been used about the father too. [In fact here, we have difficulty with translation of the mentioned phrase; 'who bore them' is translation of *"valadnahom"*.] An example of such interpretation is *"by the father and him whom he begot"* (22), which means something other than giving birth to a child and shows that both father and mother are involved in the creation of the child. Moreover, in the Holy Qur'an, Traditions and the words of Islamic jurists, giving birth to a child is instead of the phrase 'childbirth' (28). Also, in verse 36 of Surat Ale-Emran the phrase "giving birth to a child" has been repeated three times, and in all of them we have: *"and when she (Mariam's mother) bore her she said, my Lord I have borne a female (child) and Allah knew better what she had borne …"* (15). So these scholars believe that the mother is not just the woman who gives birth to a child.

On the other hand, the followers of the first opinion believe that scientifically, a child is not the product of just the ovum and qualities inherited from the woman, but the child and in general human beings are formed by their interaction with surroundings and circumstances; when it is attached as embryo to the wall of the womb, its existence as well as its mental and physical growth is influenced by the womb (22). Thereby, is it fair to call the ovum owner the mother but lower the role of the owner of the womb as the person who grows the child? (29) Thus the supporters of the first opinion, by this question make clear that only the owner of the womb is the mother. Also they rely on the experiences of some countries, as in the majority of countries including U.S.A, where the womb owner is validated as mother. So, in different states like Virginia, Arizona and Georgia the law stipulates that: if a child is born from a mother who is an embryo applicant for whom donation has been conducted with the consent of her husband, and this fertilization is performed either by donated sperm or ovum, she and her husband are the legitimate parents of the child. In another part of this law we read: "a woman who gives birth to a child is his legal mother" (30).

Therefore, this first opinion ignores the effect of the ovum owner mother on the creation of a child whereas I believe that such a child has two mothers: the ovum owner and womb owner, which it is the basic idea in the article. And this is explained as the third opinion.

### Second opinion - the owner of the ovum is the real mother

Some lawyers state that: "what is certain is that in this case legal lineage is not realized between the woman who has carried the child during its growth in womb and the child. Because the ovule of the carrier woman has had no part in the creation of the child and the growth termination in womb does not realize lineage. Therefore, the child is only connected to the owners of ovule and sperm…" (10). Stipulating the effect of the carrier on the genetic qualities and some lineage commandments, these lawyers believe that in this case lineage and inheritance exist between the owners of the embryo (31).

Some Islamic scholars consider motherhood, based on common understanding, the same as fatherhood, and believe that: "the first stage of child creation is from mother’s ovule. Thereby, when it is assumed that zygote of this child as its first element has been made possible by sperm and ovule, this first creature is the first stage of its creation. Therefore, the first stage of child's creation is formed by the owners of sperm and ovule. And even zygote of the child constitutes two combined elements, each of which is dependent on the owners of sperm and ovule. But nutrition of this child, which happens after this stage, is only effective in his growth" (14).

Regarding the role of child nutrition in womb, the scholars indicate that such nutrition does not put the child out of the state of being a child for the owners of the sperm and ovum. They continue: "thereby, as artificial formation of zygote is performed in a specific place till the spirit can be penetrated in it, and it gains the ability to live like other children out of such artificial place, the child is the child of sperm and ovule owners. If instead of such artificial platform, womb is regarded as the growth environment for zygote; such child belongs to the owner of ovule. As common's view always considers the owner of ovule as the mother of the child" (14).

Emphasizing the existence of a legitimate lineage for such a child, some believe that the child's mother and father are the man and woman who own the sperm and ovum. To them such commandment is subject to the condition that man is specified and if not, the child is only attributed to the woman who has carried the embryo in her womb and has given birth to him (32). Some other lawyers liken this issue to plants by relying on Article 33 of the Iranian Civil Code which provides that: "products and crops which have come out of the ground are the property of the owner of the land, whether their growth is natural or the result of the owner's operations, unless the product or crop has sprung from the roots or seeds of another party. If this is the case the trees or crops shall be the property of the owner of the roots or seeds, even if they have been sown without the approval of the honor of the land." believe that "disregarding the origin of child creation, which is man's sperm and woman's ovule and just considering lineage through the growth place of embryo is difficult" (33).

Therefore, with regard to embryo donation based on the second theory, the resulting child belongs to the owners of the sperm and ovum, even if the embryo is located in the womb of the applicant woman.

### Third opinion-both the owner of the ovum and owner of womb are the real mothers

According to this theory, both the owner of ovule and the owner of womb can be legal mothers of the child. In fact, contrary to the mentioned two theories, this theory emphasizes two fundamental points involving the creation of a human being. Although some verses and professional ideas indicate the role of the ovum in the creation of a human being, we cannot consider only the role of the woman's ovum. As we know, it is a scientific fact that a woman plays two roles in reproduction: with her ovum and her womb. It seems that a malfunction in each component makes reproduction impossible. Therefore, if the ovum is from one woman and womb is from another woman, they can both be regarded as mothers (34). Some of the scholars, as Mohammad Mersi, argue that: "The real mother undertakes three stages for child creation: ovule insemination, fertilization, and giving birth to the child. Undoubtedly a woman who passes such stages is the mother but the things which are evident on the surface are pregnancy and childbirth" (29).

Imam Khomeini, as an Islamic jurist, believes that if after embryo formation in the mother's womb it is transferred to the womb of another woman, such transfer can happen before the breathing in of spirit or after it. If such transfer happens after breathing in of spirit, the child belongs to the first woman. But if transfer happens to the womb of the second woman at the time of being clinging mass and fleshy tissue (i.e., the fetus in the early stages of its development) or generally before completion of creation and breathing in of spirit, the issue of lineage becomes more challenging. If it is proved that the zygote resulting from a certain couple is the origin of such child, the child belongs to the couple, whether it is transferred to a natural womb or an artificial womb (35). And since the embryo transfer usually happens before the completion of creation, this observation might confirm our accepted theory.

Some scholars believe in considering two mothers for such a child, and indicate that in this case, custom establishes the lineage between the sperm owner and the child. Therefore, the legitimate father of the child is the sperm owner and the wife of the sperm owner has no relation with the child, as she has had no part in the physiological aspect of child creation. But a mother and child relationship exists between the child and the two women (i.e. the ovum owner and the carrier of embryo). Thus, this view neither considers the ovule owner as the only mother as Imam Khomeini says, nor only the owner of womb as according to Ayatollah Khoei or Ayatollah Araki. Yet based on the mentioned view having two mothers is not problematic, what is impossible is having two fathers not two mothers; moreover, custom establishes these relationships with two mothers" (36).

Although accepting two mothers is difficult as it is not normal, it seems that the third opinion that recognizes both ovum owner and womb owner as the mothers of a laboratory child is more effective regarding motherhood lineage because both women play roles in the creation and development of the child. Therefore, preferring the influences and role of one of them compared to the other and attributing lineage to one of them requires scientific reasons. Moreover, it can be said that motherhood is determined by professionals in this regard. In other words, motherhood in view of medical science is subject determination and as it is said, subject determination is not the responsibility of the Islamic jurists. In view of medical science, it is evident that the origin of the cell which creates the embryo on the mother's side, is a woman's ovum. But based on the latest medical knowledge, the womb plays various important roles in nutrition, growth and embryo support.

Here, a question may arise that in the third opinion the problems of confidentiality of information related to the donated embryo, especially from the mother's side, are ignored. However, this problem concerns only the proving aspect and does not cause a loss to actual aspect (or causality relationship). Although we accept that there are some problems regarding the confidentiality of related information, it is important from the proving aspect to realize the lineage for its consequences, such as marriage, which should not happen between non-marriageable persons. And the lineage realization in the mentioned case in both motherhood and fatherhood lineages is necessary.

According to the author, the two mother system theory is preferred to the other theories, because with regard to the lineage issue it does not involve the problems of the other theories. This theory does not ignore the genetic and carrying factors. In other words, this theory is a comprehensive theory, since it considers the first stage and the origin of child's creation i.e. a woman's ovum as well as the effect of the womb on the child growth and nutrition and also childbirth.

### Part 2: pre-implantation embryo’s inheritance

'Inheritance' means property or right which is delivered to the survivors after a person dies (37). This concept in legal terms means non-contractual transfer of the property of a deceased person to his heirs (38, 39). Inheritance is one of the legal outcomes of relationship by blood. Absolute condition of inheritance based on Iranian Civil Code Article 861 is lineage. Thus, it can be said that a child resulting from non-adultery is entitled to receive inheritance only if lineage is established. Therefore, all other types of offspring enjoy the state of a deceased of their devisor including the pre-implantation embryo or laboratory child, who is considered as descendant.

Some issues arise about inheritance in the area of embryo donation, among which we study the most important ones: As inheritance between pre-implantation embryo and the genetic father, inheritance between laboratory child and the genetic and carrier mother, and the starting time for the pre-implantation embryo to be entitled to inheritance rights.

### Inheritance between pre-implantation embryo and the genetic father

Undoubtedly, under inheritance conditions, the pre-implantation embryo inherits from his genetic father, because lineage is realized from child creation through another gamete. In this case, production and genetic relationships exist between the child and genetic father. Consequently, inheritance exists between them.

In view of most Shiite and Sunnite jurists, whenever *in vitro* fertilization is performed by the husband's sperm and the ovum and womb of the wife or other wife of the husband, the child is attributed to the owner of the sperm (i.e. the husband) and the woman who owns the ovum or the womb (based on different opinions); therefore inheritance is established between them. As mentioned earlier, among Shiite jurists only Ayatollah Hakim has pronounced a judgment about the improbability of child attribution to the owner of the sperm (i.e. the husband) and considers the child connected to the mother (40). In conclusion, in this view inheritance between the laboratory child and the owner of the sperm is not established.

Regarding birth through *in vitro* fertilization, Islamic law stipulates that (30, 41) due to complexities of legal issues in this area, financial problems are solved through compromise. But according to the fact that in embryo donation the lineage is realized, as indicated in the article, and the condition of inheritance is a legitimate lineage, using the suggestion of 'compromise' to solve the problem of inheritance of a child resulting from embryo donation as suggested by some lawyers, is questionable (42). That is, firstly because inheritance rules are mandatory rules and its contrary cannot be consented mutually. Secondly, the suggestion for compromise has not specified action in cases which lead to the lack of compromise.

Thereby, as the author believes, the pre-implantation embryo, after lineage is proved and based on general conditions of inheritance, inherits from his genetic father. If it is stipulated that the identity of the genetic father is not clear, and proving lineage is not realized; then inheritance between them (child and genetic father) is out of question (43). But to explain this problem, it can be said that: according to Article 1 of the Conditions of Embryo Donation to Sterile Couples Act, year 2003, and based on the phrase "after the written consent of embryo owner couple…" , the identity of genetic father is clear for fertilization centers. However, according to the fourth paragraph and also the annex of Article 6 of regulations issued in 2004 in relation to this Act, receipt, maintenance and transfer of donated embryos must be performed under totally confidential conditions, and the information related to the donated embryos must be classified as totally confidential. Furthermore, with the assumption that the identity of the genetic father is not clear due to the confidentiality of information related to this, from theoretical aspect of inheritance bases the inheritance right, for attributing lineage to the genetic father, conforming to reality is not rejected. Although from a proving aspect it may be difficult unless such information is available for donors and recipients in such institutes, and this makes the problem even more complicated.

For example, a pre-implantation embryo, developed into a person with an independent personality, believes that the man whom he has known since the first day of his life is his father and the woman who has given birth to him and breastfed him is his mother. When faced with the inheritance issue, involving attributing his lineage to his genetic father, and on the other hand, having the relation between himself and the embryo recipient couple discounted as lineage, this person confronts a difficult and complex problem which has serious mental consequences for him.

Maybe that is why the law of some countries like France, contrary to their traditional rules which say that a child must be attributed to the sperm owner, stipulates that if the embryo results from a third party of unknown identity, it must be attributed to the applicant couple not the sperm owner. In this case, the sperm owner has immunity regarding bringing an action for tort (37). Although such a solution can solve a lot of future problems for the pre-implantation embryo including lineage, inheritance, guardianship, maintenance and so on, and the child faces to only one legal or commissioning couple as father and mother, using the experience of other countries must be considered subject to religious, moral and cultural aspects of our country.

It must be noted that if we cannot or do not want to prove lineage between a pre-implantation embryo and genetic father and mother with regard to the complex legal and the child's mental problems, is it possible that such a child enjoys the inheritance from the commissioning couple? (When lineage realization and as a result, inheritance between them, is rejected). Some thinkers suggest that "after gamete donation and formation of zygote in womb under another irrevocable contract, sperm or ovule receivers accept the child custody and maintenance and promise to mention in their wills that this child is like their first class heirs, so receives one-third of the property. And such will as an obligation of irrevocable contract is enforceable" (15). Though this suggestion seems to solve the case, in practice concerning other heirs this theory cannot eliminate the problems.

In conclusion, in view of general principles of law, since lineage is realized between the laboratory child and the genetic father, the inheritance exists between them, although practical application of this law is fraught with problems.

### Inheritance between laboratory child and genetic or carrier mother

Inheritance between child and the genetic or carrier mother is dependent on the proof of motherhood lineage and this issue differs according to different bases in determination of the genetic mother or carrier mother (commissioning mother) as real mother. In this regard, three theories are considered.

1. If the genetic mother is known as the only real mother of the laboratory child, inheritance is realized only between child and the genetic mother. Therefore, no lineage is established between such a child and the carrier or commissioning mother so inheritance is not established. If we consider the carrier mother as the foster mother (28, 44) from this follow all the rules concerning foster mothers, including prohibition of marriage to the child. As stated earlier, this theory has its own followers (10, 28, 30, 39, 45). But in the opinion of the author of this article, the genetic mother is not the only mother of the laboratory child.
  The important issue here is how it is possible to realize practically the inheritance between the genetic mother and the child, because if the identity of the genetic mother is not clear for any reason, inheritance is not realized as lineage to this mother is not provable to. But if the identity of this mother is clear, proving the lineage attribution is possible, and inheritance is established. But the same problems stated about inheritance between laboratory child and the genetic father, exist here too.
2. If we consider the carrier mother as the only mother of the child, the inheritance relationship between the child and this mother is established after lineage between them is accepted. Even if this woman is not the wife of the sperm owner, since inheritance exists just after proving the lineage relationship. This theory has its own followers too (46) and therefore, realizes inheritance between the carrier mother and child. Although such theory solves the future problems of the pre-implantation embryo in terms of inheritance and lineage, and also does not have the problems that other theories have, accepting that from a medical aspect the ovum owner, who has an important role in the creation of the pre-implantation embryo, is not his mother, becomes difficult. Moreover, this theory has attracted serious opposition from the Islamic and legal laws.
3. If both genetic mother and carrier mother are considered as real and blood mothers of the laboratory child and attribution of lineage to both of them becomes possible, inheritance is realized between them too. This theory also has its own followers (47). But some Islamic jurists believe that these two mothers are as the foster mothers of the child (48) so based on the legal principles there is no inheritance between them. If the child has two real and blood mothers, it should be examined in the first place whether it is possible that a person inherits from two mothers or two mothers inherit from one child. If the answer to this question is yes, how much is their share of inheritance?

In this case if the theory of having two mothers is proved there will be no obstacle to their inheritance (49) because the child's share of inheritance from two mothers is determined based on inheritance law. According to Article 865 of the Iranian Civil Code; "if several causes of inheritance are united in the same person, he takes inheritance from all the causes". Therefore, "everybody who meets each sort of relationship stipulated in the Civil Code, which is the requirement of inheritance, receives his estate of a deceased based on his relationship; because the Iranian law has recognized each one of the relationships as the requirement for inheritance" (33). Although to inherit from several causes includes a combination of relationships by blood or relationships by blood and marriage, it includes the subject in question too, since several causes of inheritance (inheritance from two mothers) have been united in one person who is a laboratory child (50).

In answer to the question of how much is the two mothers' share of inheritance from the laboratory child, some Islamic jurists believe that each mother receives a share of inheritance as follows: in the event of such a child not having children one third of the estate of the deceased is mothers' share and is halved between them (51). This is because both two mothers have been effective in the creation and growth of the child and there is no reason to prefer one of them to the other. However some jurists refer to the superficial meaning of the 11th verse of Surat Al-Nesa which reads: *"but if he has no children, and his heirs are his parents, a third to his mother"* (3) and indicate that the mother’s share in case of not having any children is one third of the estate of the deceased and if we accept the theory of having two mothers then we have to consider a share of one third for each of them, which is not fair regarding the shares of other heirs (41).

For criticizing the last view, it can be said that firstly, one third share for the mother has been considered in most cases and the legislator has issued this rule generally, in normal conditions where every child had one mother. But the legislator was not trying to consider special cases (having two mothers), otherwise it should be noted clearly, and the superficial meaning of the verse says nothing of exceptional cases. So it does not seem fair regarding such special cases, to refer just to the superficial meaning of the verse, which is justified in normal situations. Secondly, according to the author of this article, a share of one third is for the title of 'mother' in comparison to the title of 'father', 'wife' or 'husband' whether one mother or two. Therefore, through one third shared between both mothers no damage is made to the shares of other heirs. Thirdly, the rule of halving (i.e., equity between two persons) in the majority of cases in which there is no decisive reason for the amount of shares of the parties is applicable. From our discussion we know that two mothers have been effective in the creation of the child and therefore receive a share of inheritance from the child for their title as mothers, and we also know that there is no reason to prefer one of them to the other, so the halving rule is the fairest way of treatment. In fact, with the assumption of having two mothers, no other heirs lose their shares, and also the two mothers receive equal shares.

Therefore, the accepted theory of the author regarding having two mothers on the one hand respects the mandatory rule of inheritance and on the other hand based on facts and emerging issues in society has made use of the fair principle of halving. As mentioned, some Islamic jurists have also introduced this theory (46). And some others (30, 36) have suggested compromise between the heirs in the case of a laboratory child, despite the fact that inheritance rules are mandatory rules, which might be the result of paying attention to the facts and new problems of society. In conclusion, in view of general legal principles and based on realization of lineage between laboratory child and both the genetic mother and carrier mother, inheritance between them is established even if this causes practical problems.

### The starting time for the pre-implantation embryo to be entitled to inheritance rights

Article 957 of the Iranian Civil Code which lays down: "A child in the womb will enjoy civil rights provided that it comes into the world alive", considers the condition for an embryo to be entitled to civil rights within a general rule. The condition stipulated in this Article is also repeated in Article 1270 of the Iranian Civil Code which states: "A confession made in respect of an unborn child is only valid when the child is born alive". According to what has been stipulated in these Articles, establishment of civil rights including inheritance is conditional on the safe birth of the child and this causes to be found out that child has had such competence since the zygote formation and during pregnancy. There is no doubt in terms of application of such rights to the fertilized ovum in the womb or the embryo resulting from in-vitro fertilization from the time of its transfer to the womb because the embryo is subject to receiving some rights including inheritance according to Islamic law and related legal rules. The question here is whether an embryo can enjoy inheritance rights before being transferred to the womb, as after 'being born alive' such rights could be established for it since the period of growth out of womb. In other words, when is the pre-implantation embryo entitled to enjoy inheritance rights?

Though it is true that civil rights including inheritance rights are legally accepted for the embryo and customarily, embryo refers to the one creature developing in the womb; such a title is not applicable to the embryo as long as it is out of the womb. But considering the true meaning of the mentioned rules it is concluded that the real intention of the legislator is to recognize such rights for the embryo as a potential human being, whether in-vitro or in the womb. The main focus in the rules related to the embryo is the potential human being, even if it is developing out of the womb (10). But some other lawyers believe that the subject of jurisprudential and legal rules is the embryo, and referring to this subject in medicine by the zygote formed in-vitro (out of womb) in relation to the influence of jurisprudential and legal rules is not enough (52). Mentioning the questionable using of embryo as the 'zygote formed' in-vitro by custom, some scholars indicate that such doubt is an indicator of this fact that the word embryo has not been devised for the zygote formed in-vitro, and it also indicates that this pre-implantation embryo does not have any inheritance rights (41).

In the view of the author of this article, embryo is also used to refer to the pre-implantation embryo formed before transfer to the womb because limiting or expanding any concept requires referring to the particular custom. Here the judgment of particular custom such as medical custom is the criterion for realization of the concept. In physicians' view, a zygote is the result of the act of mixing a fertilized ovum with in-vivo tissue (47). Therefore, in view of this definition, the word embryo is also referred to the zygote formed in-vitro and its location in the womb is not counted. So, the pre-implantation embryo enjoys inheritance rights, but we shall separate two different cases:

1. When the formed zygote continues its growth without freezing, whether in the womb or out of it (53). In this case the embryo enjoys inheritance rights and after his safe birth it is realized that he has started having civil rights including inheritance rights since the time of embryo formation, and continues to keep these rights after being born alive.
2. When after formation the embryo is frozen and its growth stops. Under these circumstances, as a result of not being in the direction of growth and development, the embryo has no inheritance rights till its growth starts again.

Finally, another condition on the inheritance of the embryo is briefly discussed: according to Article 875 of the Iranian Civil Code, which prescribes: "It is a condition of inheritance that the heir should be alive at the moment of the death of the person from whom the inheritance issues; and if it is a question of an unborn child, it takes an inheritance only if it was conceived at the moment of death, and if it was born alive, even if it dies immediately after birth" the condition of embryo inheritance rights is its formation before the moment death of creator and also the safe birth of the child. Therefore, based on the fact that inheritance is one of the civil rights, it can be concluded that the inheritance rights of the pre-implantation embryo start since the formation of embryo on the condition that it is born alive. It can be deduced from the above Article that if at the moment of the death of its creator the embryo is not formed by any reason, the inheritance will not be established and so the embryo receives no share of inheritance from the creator (33).

## Conclusion

An accepted definition of lineage in this article is the creation of a human being from the gamete of another human being. The pre-implantation embryo's legitimate lineage is realized as one of the mentioned cases considered, out of a family bond among the persons who play role in its creation. Such insemination is included in Article 1158 of the Civil Code, and presumption of legitimacy is applicable to that too. Thus, the sexual intercourse of the couple in a general sense includes both the natural form and artificial insemination.

Based on medical science, jurisprudential rules and also common understanding, undoubtedly the origin of the embryo on the father's side is sperm. But regarding the determination of the motherhood lineage, there are different opinions:

The followers of the first opinion, by relying on a Qur'anic verse believe that only the woman who gives birth to a child is the mother. Others' interpretation of the verse shows the phrase used in the verse does not mean giving birth to a child, as in some Traditions (Hadith) this phrase has been used about the father too; yet it means something other than giving birth to a child and seems to convey that the father and mother are the origins of the child. The use of this verse by the followers of the first opinion is that this verse only considers a woman who gives birth to a child as mother, whether the ovum is hers or not. On the other hand, science supports this view in that a child is not only the product of the ovum and qualities inherited from its owner, but the child and in general human beings are the result of their interaction with surroundings and circumstances. Also, this group relies on the experiences of some other countries. But the author of this article has found no evidence that only the womb owner is the mother of a child, because the first stage of child creation formed by the ovum owner is disregarded in this opinion.

Stipulating the influence of the carrier mother on the genetic qualities and some lineage commandments, the lawyers, based on the second theory, believe that in this case a legal lineage exists between the embryo and the owners of the sperm and ovum. Since it is assumed that zygote of this child as its first element has been made possible by the sperm and ovum, therefore the first stage of the child's creation is formed by the owners of the sperm and ovulm. But the nutrition of this child, which happens after this stage, is only effective in his growth, and such nutrition does not put the child out of the state of being a child of the owners of the sperm and ovum. This opinion likens the carrier mother's womb to an artificial platform regarded as the growth environment for the zygote. Also they believe that common view always considers the owner of the ovum as the mother of the child. Yet to some of believers of this theory, such commandment is subject to the condition that the father is identified and if not, the child is only attributed to the woman who has carried the embryo in her womb and has given birth to it. But since the second opinion considers only the first stage of child creation, which is formed by the ovum owner, the author believes that this opinion is incomplete.

The third theory, which is selected by the author, emphasizes two fundamental factors involving the creation of human beings: the genetic and carrying factors. Although some verses and professional ideas indicate the role of the ovum in the creation of a human being, general custom considers the one who has carried embryo in her womb and has given birth to it as mother of the child. It is evident through scientific advancements that a woman plays two roles in reproduction: her ovum and her womb. It seems that a malfunction in each component makes reproduction impossible. But out of these two essential roles, the thing which is evident on the surface is the role of the woman's womb i.e., pregnancy and childbirth. Also, some Islamic jurists that believe if the transfer of the embryo happens to the womb of a second woman, before completion of creation and breathing in of spirit, the issue of lineage becomes more challenging, and this transfer usually happens before completion of creation, so this observation might confirm our accepted theory.

Although accepting two mothers is difficult as it is not normal, it seems that the third opinion that recognizes both ovum owner and womb owner as the mothers of a laboratory child is more effective regarding motherhood lineage because both women play roles in the creation and development of the child. Therefore, preferring the influences and role of one of them compared to the other and attributing lineage to one of them requires scientific reasons. Moreover, it can be said that motherhood is determined by professionals in this regard. And in view of medical science, it is evident that the origin of the cell, which creates the embryo on the mother side, is a woman ovum. But undoubtedly, based on the latest medical knowledge, the womb plays various important roles in nutrition, growth and embryo support. So general custom concerning determination of motherhood lineage is questionable and common judgment is not reliable.

Thus, according to the author, the two mother system theory is preferred to the other theories, because with regard to lineage it does not involve the problems of the other theories. This theory does not ignore the genetic and carrying factors. In other words, this theory is a comprehensive theory, since it considers the first stage and the origin of child's creation i.e. a woman's ovum as well as the effect of the womb on the child growth and nutrition and also childbirth.

In relation to the pre-implantation embryo's inheritance, undoubtedly, under inheritance conditions, it inherits from his genetic father, because lineage is realized from child creation through another gamete. The author believes that with the proof of having two mothers, no obstacle is in the way to establish inheritance, since according to Article 865 of the Iranian Civil Code, if several causes of inheritance (inheritance from two mothers) have been united in one person (the laboratory child) he takes inheritance from all the causes.

But in answer to this question of how much is the two mothers' share of inheritance from the laboratory child, some Islamic jurists believe that each mother receives a share of inheritance halved between them. The accepted theory of the author regarding having two mothers on the one hand respects the mandatory rule of inheritance and on the other hand based on facts and new problems of society, has made use of the fair principle of halving.
